# Presto lift—a facelift that preserves the retaining ligaments and SMAS tethering

**DOI:** 10.1007/s10006-016-0594-x

**Published:** 2016-12-02

**Authors:** Wolfgang Funk

**Affiliations:** Clinic for Plastic, Aesthetic and Reconstructive Surgery, Frau-Holle-Straße 32, 81739 Munich, Germany

**Keywords:** Rhytidectomy, SMAS technique, Retaining ligaments, Facelift, Esthetic surgery

## Abstract

**Background:**

Producing youthful facial appearance by face-lifting often comes along with an undesired loss of patient’s individual phenotype. This may result from insufficient preservation of retaining ligaments, the “guardians of facial identify,” and from severance of the intersegmental connections of the superficial musculo-aponeurotic system (SMAS), which tether, structure, and compartmentalize facial soft tissue into defined, relevant anatomical zones.

**Methods:**

The technique reported here preserves most retaining ligaments. They serve to fix the facial soft tissue mass in loco. With the possible exception of the zygomatic-cutaneous ligament, they are only carefully distended. The SMAS intersegmental connections and the zygomatic SMAS border are preserved to retain effective points of facial tissue fixture. Aging-associated thinning and lengthening of the lower eyelid are reduced by midfacial-submalar preparation (Aston 1996). Subplatysmal preparation and disconnection of the cranial-platysmal border permits optimal modeling of neck structure.

**Results:**

The combination of preservation of retaining ligaments and SMAS tethering (“PRESTO facelift”) introduced here as a novel face-lifting technique conserves the individual esthetics of the patient by approaching her/his individual phenotype from decades ago. In addition, undesired outcomes of facelift surgery and common risks of facelift surgery are circumvented.

**Conclusions:**

The PRESTO facelift technique generates optimal esthetic results that conserve a patient’s personal facial identity, besides restoring a more youthful appearance and being rapid and safe.

## Introduction

Facelift surgery is challenging with transforming an aging face into a desired, more youthful facial appearance [1, 2]. Sometimes struggling between transformations toward a “nameless beauty” phenotype and conserving the patient’s personal identity. The outcome should be predictable, effective, long lasting, and easy to learn and to apply.

A retrospective study of more than 8000 cases postulates the relation of morphological characteristics and natural appearing results [3]. Patients’ identity and the surgical treatment and technique for facial rejuvenation are dependent on each other and should support each other. We want to present a technique considering both. The basics of some of the actual techniques are a wide undermining of the skin, the superficial musculo-aponeurotic system (SMAS) [4] manipulation in the face and neck area, including a pull to each layer cranial—dorsally. Other techniques are doing a wide skin undermining and plication of the SMAS [5]. Most of these standard techniques have their focus on the techniques and the anatomical situation.

Preservation of the individuality of a facial expression—despite surgical intervention—can be missed by well-accepted face-lifting techniques. They can induce one or several of the following undesired outcomes: for example, over- or undermodelling of phenotype-determining facial structures leading to alienation, lateral overstretching of the oral contour, insufficient midface lifting, overmodelling of midface structures, unnatural voluminizing of the cheeks, and/or mask-like overall facial appearance. Together, these undesired outcomes threaten the conservation of a patient’s individual facial expression and phenotype over time [6]. Hills [7] reported about the importance of face identity and the adaptation by others and Hamra [8, 9] published on the unhappy signs of surgery and that conventional facelift techniques are not predictable and are often unfavorable. Stuzin [10] concludes that a patient’s specific plan is mandatory related to the artistic goals of the surgeon; he postulates his technique for malar augmentation and facial width; this implements a more anatomical than identity approach. Castello [11] reviewed 327 patients with a modified superficial musculo-aponeurotic system facelift; his concern with the conventional SMAS facelift was the skin laxity and sagging tissue. He used a patient satisfaction questionnaire, which was either sent to the patient or by a telephone interview. Swanson [12] did an outcome analysis in 93 facial rejuvenation patients with a 96, 7% satisfaction rate that the result meets patients’ expectations.

In summary, facelift requirements are durability, naturalness, comparability to the patient’s younger face = identity, safe technique (minimized complications, easy to adopt), reproducible, predictable, and effective. The technique should enhance the balance between effectiveness and identity conserving.

The focus of this new technique we are presenting is on the patient’s side to maintain the identity after the surgical rejuvenation [13] and on the surgical side the maximal mobility of the forming anatomical structures and the minimal possibility of overdoing and defacement. The SMAS has omnipotent shaping features and the retaining ligaments are the fixation elements of the complete soft tissue bloc, due to their osteo- and fascio-cutaneous stabilizations. The presented technique preserves the interseptal fixating structures of the fat compartments, the retaining ligaments, and the cranial and the cranio-dorsal SMAS borders. The technique permits a complete subplatysmal preparation, a mobilization of the SMAS, a dissection plane, as described by the extended facelift of Hamra [14], and a distension of the infraorbital, ligamental area. Our objective in this study was to describe and evaluate the Presto facelift.

## Methods

### Characterization of the patients

The author with informed consent and under general anesthesia performed PRESTO-facelift surgery. Between March 2009 and February 2010, 46 female patients, aged 42–68 years old (mean 51.5 years), were treated with PRESTO-facelift technique and were evaluated with the ANA-scale. All patients were in an age-based health condition. The nutritional condition was in the normal range (BMI 21, 5–28/mean 24, 3). None of the patients had a surgical relevant additional disease. Notably, no diabetic or anticoagulation relevant patient or strong smoker (more than 20 cigarettes/day) were included in the study group.

### Surgical technique

**Fig. 1:**
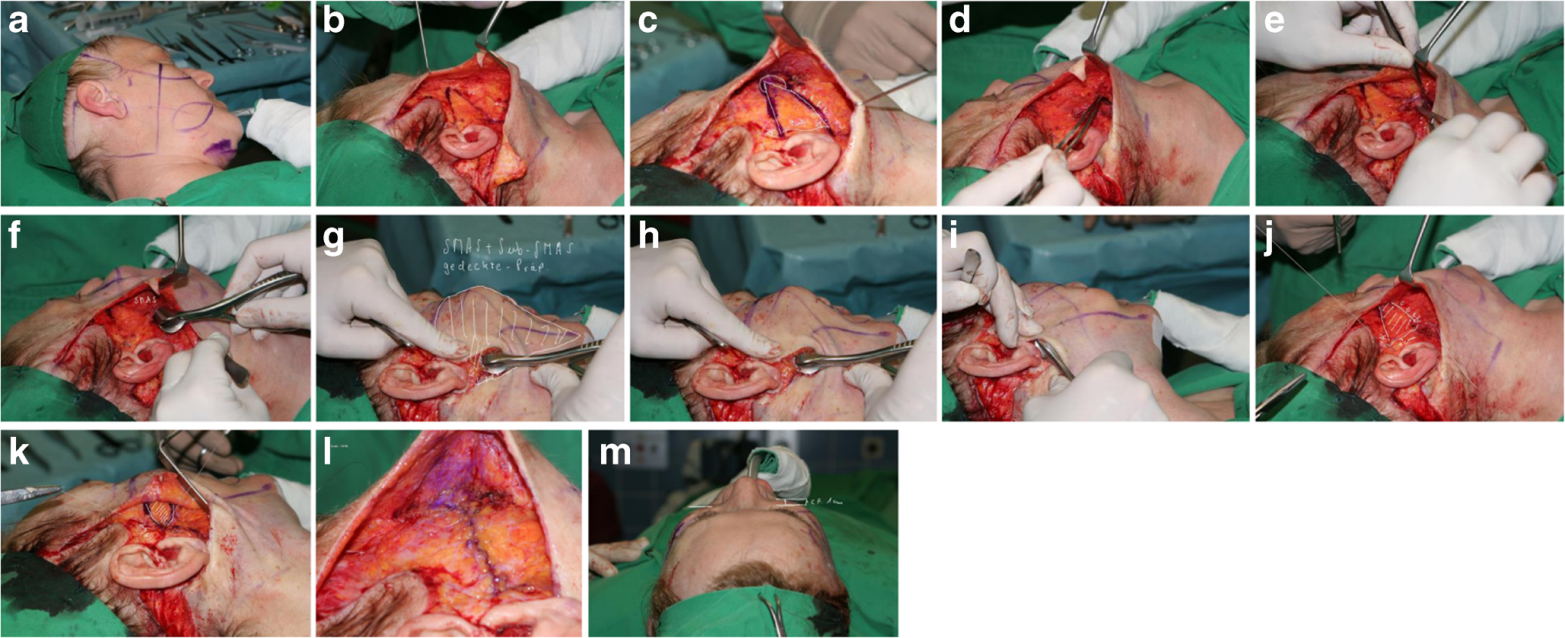
**a** Marking of the subcutaneous preparation and the incision line**. b** Marking of the dorsally open triangle with the cervical wing. **c** Marking of the dorsally open triangle with superposed circle and marking of the vectors. **d** Entry to the SMAS and subSMAS. **e** Subplatysmal blunt dissection in the cervical wing. **f** SMAS and subplatysmal preparation. **g** Segmental subSMAS preparation reaches from the zygomatic bone to the malar area, from the malar area under the nasolabial fold to mouth angle and over the mandible jaw line to connect the facial and the cervical plane. **h** The subSMAS introduced instrument shows the freeing from about 2 ½ cm in heights of the SMAS and fat compartment complex. **i** Subplatysmal introduced instrument points to the corner of the mouth with dissected cervical transition. **j** After preparation of the rotation area with approximation of the wings A and A’. **k** Approximated endpoints A and A’ with a lifting capacity of ∼4 cm. **l** Anatomical view of the fixated and cranially rotated area. **m** Side comparison between operated (*right*) and non-operated (*left*) side focusing on the voluming effect in this case of ∼1 cm

**Fig. 2:**
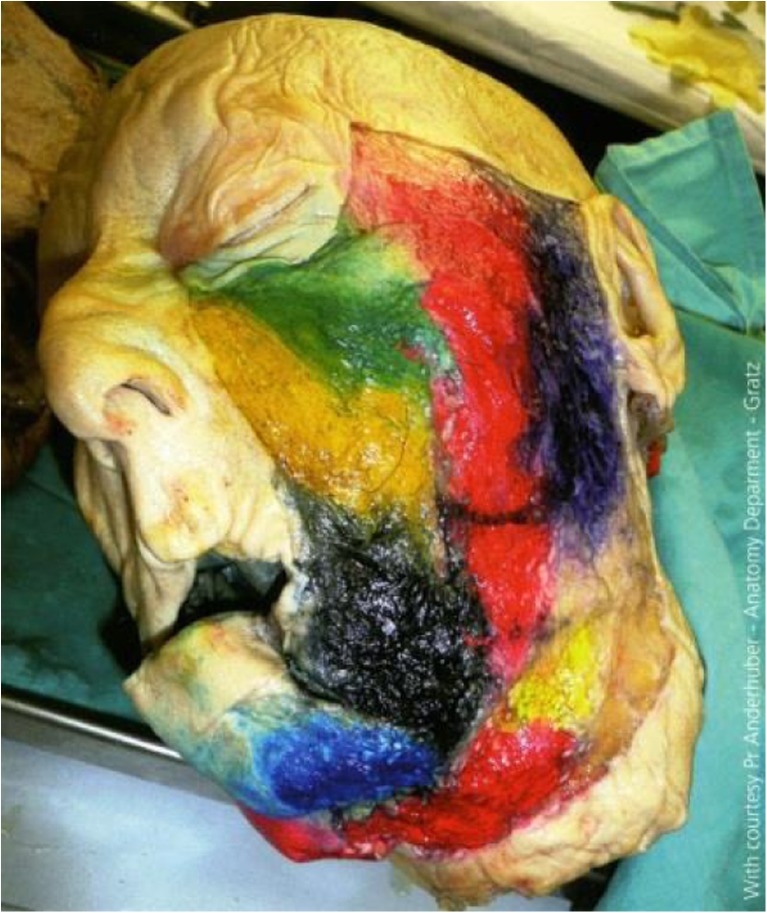
Midfacial and lateral esthetic relevant fat compartments (with courtesy of Prof Anderhuber—Anatomy Department, Graz)

The primary incision of the skin begins at the caudal aspect of the temporal hairline, extends to the superior pole of the ear then caudally along the contours of the ear and the tragus (preferentially pretragal) (Fig. 1a). The preparation continues by circumscribing the external ear and proceeding into the hair of the occipital region. Subcutaneously, the medial boundary is the superior margin of the orbit, which continues up to the end of the first third of the eye socket and then caudally to the mandible about 3, 5 cm distant from the earlobe. From there, it passes further into the region of the neck approximately 2 cm below the punctum nervosum. From there, the preparation extends into the occipital region and over the mastoid. Here, care must be taken to ensure that the blood supply via the subcutaneous layer is maintained. After skin preparation, an approx. 5 cm line extending from the mandibular angle is followed caudally into the neck region (Fig. 1b). In the cheek region, a triangle is marked which is open posteriorly and whose upper side precedes approx. 1 cm above the zygomatic bone. The lower side proceeds from the first third of the zygomatic bone caudally to the mandibular angle. The marking is expanded upon using a circle of approx. 2 cm in diameter, located at the tip of the triangle, which extends over the tip of the angle by about ½ cm (Fig. 1c). The cervical SMAS-platysma complex is opened and subplatysmal preparation is carried out up to the hyoid bone and the clavicle (Fig. 1d–f). Tissue distension is performed very carefully with a blunt dissector, thus respecting and protecting regional vessels and nerves [15] land the parotid duct. The resulting detachment of this layer permits the use of cranial vectors in the cheek region without any restraint through the platysma (Fig. 1g–i). From the mandibular angle, a covered sub-SMAS preparation is carried out bluntly as far as to the zygomatic bone and over the entire region of the cheek, the maxilla [16–18] and the mandible. Hereby, the cranial and caudal fixation points of the SMAS are retained (“SMAS tethering”). In the same way, the key retaining ligaments remain intact in order to guarantee continued facial soft tissue fixation, and thus conservation of the individual facial esthetics. The overall goal is a complete laminar detachment of the individual SMAS sub-zones (Fig. 2), while maintaining SMAS fixation at its intersegmental borders [19, 21, 22]. This combination of techniques avoids integumental overextension by preservation of the individual, dynamic and anatomical boundaries, while maintaining excellent mobility (Fig. 1j). After distension of the SMAS as described above, the dorsal, caudal final point of the arm of the triangle—called A—is attached to the final dorsal point of the cranial arm—called A’—and the entire tissue block is lifted and sutured in the direction of the circle (Fig. 1k). A SMAS lifting of approx. 3½ to 4 cm can be achieved. The circular form is swung from caudal in the cranial direction into the zygomatic-orbital passage. Through this last maneuver, the optic extension of the lower eyelid is shortened so that the midface and the malar fat pad are lifted over the previously detached SMAS structures. The fixation of the SMAS is carried out using a permanent 3.0 Terylene^®^ 1 polyethylene terephthalate suture. The vector direction is cranial, with mild lateral components. The direction of traction guarantees a harmonious passage from the zygomatic bone into the lateral region of the orbit and the tissues with its anterior portions lifting the existing malar fat pad and raises it in such a manner that a voluming effect occurs (Fig. 1l). This lifting effect is protected from adopting a new, undesired shape through tethering by the preserved SMAS intersegmental structures and the retaining ligaments. The further procedure involves cervical tightening of the SMAS-platysma complex with fixation to the mastoid process. This is subsequently followed by stopping any hemorrhaging, skin resection, and layer-by-layer suture closure.

### ANA-scale parameters

Outcome was assessed by a newly developed esthetic satisfaction scoring system, the esthetic numeric analog scale (ANA-scale) [23]. The time of assessment was preoperatively (day before surgery) and 6 months postoperatively. The ANA-scale facilitates the objective, quantitative, fast, reproducible, and uniform evaluation of esthetic procedures. For the main purpose of the PRESTO face-lifting technique, it was most important to assess whether or not the patient’s personal facial characteristics over time (“individual esthetics”) had been conserved.

## Results

**Fig. 3:**
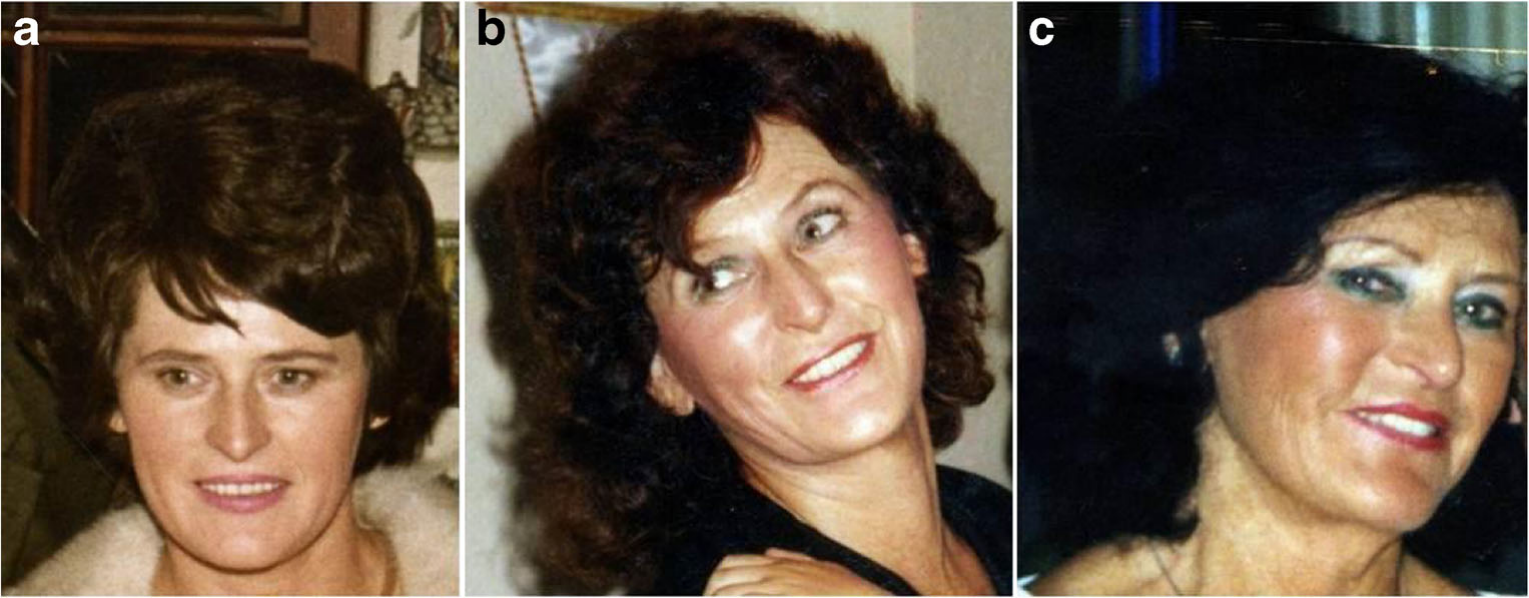
**a**–**c** Patient 31, 58, and 75 years with conserved identity after two facelifts, eye surgery, and nose surgery

**Fig. 4:**
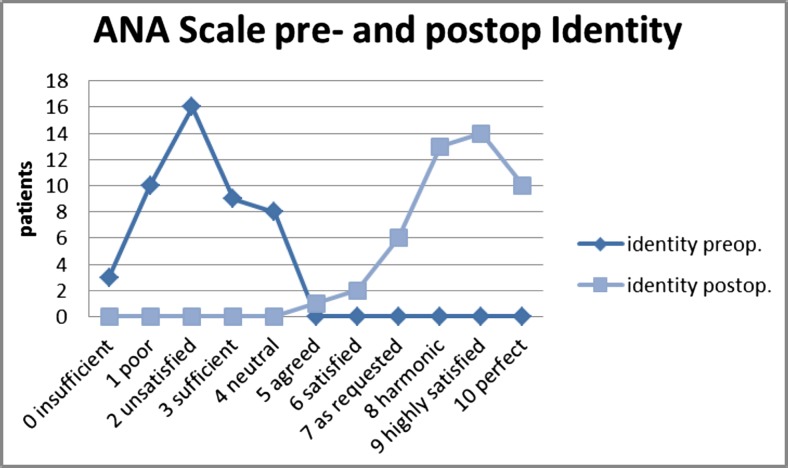
Identity conservation—the patient rates his phenotype related to her/his individual youth appearance. The patients’ perspective changed from ANA—point 2 preoperatively to ANA—point 9 postoperatively (follow-up 6 months). The ANA—delta is 7

**Fig. 5:**
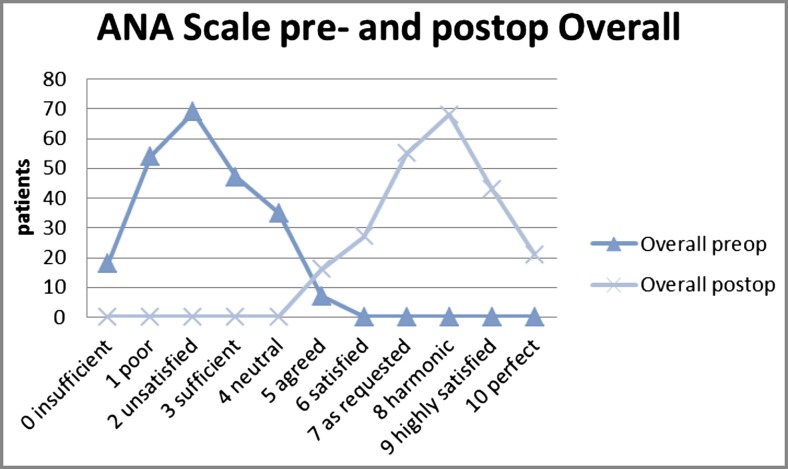
Overall patient satisfaction: changed from ANA—point 2 preoperatively to ANA—point 8 postoperatively (follow-up 6 months). The ANA—delta is 6

For validation of the outcome of technique, we have used the ANA-scale. The described technique achieves the desired lifting of the midfacial portion (Oggi line; Fig. 1m) with harmonic contouring of the midface-lateral orbital interface. Most importantly, this novel surgical approach to face-lifting not only retransforms the patient’s facial phenotype to a more youthful appearance but also strikingly conserves the patient’s personal identity [24]. Figure 3 exemplarily demonstrates a case, where this face-lifting technique has recreated a close resemblance of the patient’s former, highly individual facial physiognomy rather than a standardized beauty norm. Separately, we have quantitatively assessed how often this can be achieved with the SMAS tethering and retaining ligament preservation technique described here. Surgeon and external physician both judged patient identity as being well-preserved in all of the 46 PRESTO-treated patients. This judgment was shared by all but one of the patients (95%) (Fig. 4). ANA-scale data were complemented by additional patient-, surgeon-, or external physician-assessed subjective outcome measurements (Fig. 5). In our cohort, we had one temporary hematoma, one retroauricular prolonged superficial wound healing disorder, nine seroma, and one palsy (Table 1).

**Table 1:** Complication rate

Complications *n* = 46	*n*	^1^Percent
Infection	0	0
Wound healing	1	2
Keloids	0	0
Enlarged scars	0	0
Hematoma temporary (6 weeks)	1	2
Seroma	9	20
Palsy	1	2
Mimic disharmony	0	0
Skin dystrophy	0	0
Pigment deferral	0	0
Teleangiectasia	0	0
Salivary stone	0	0

## Discussion

**Fig. 6:**
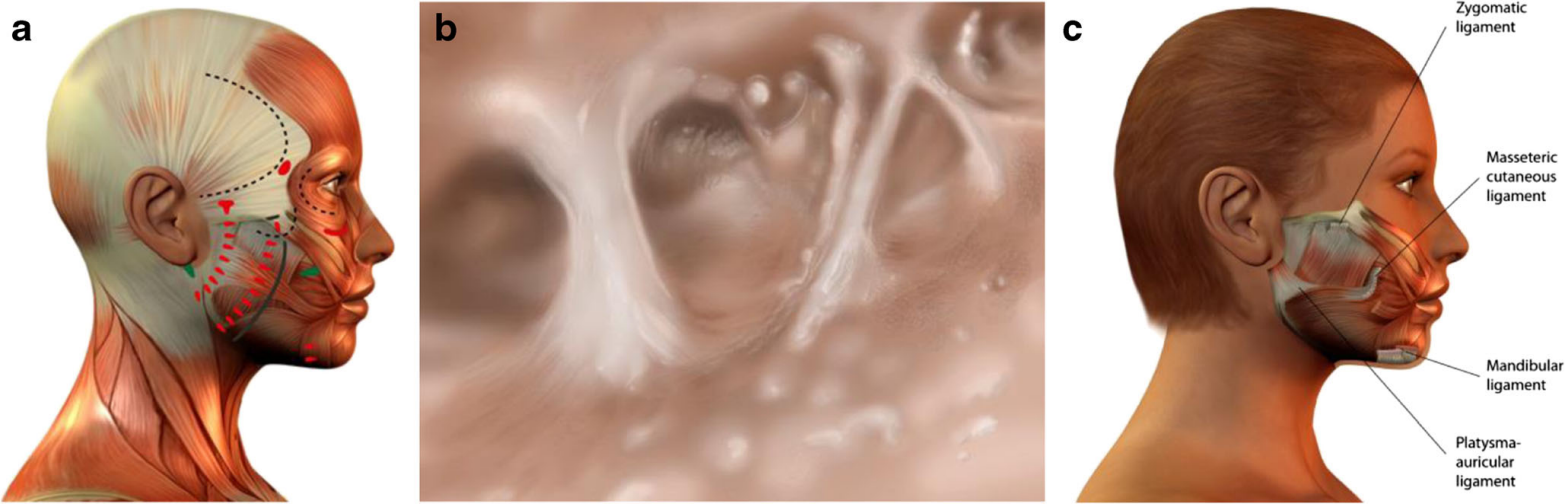
**a** Static and dynamic structures of the soft tissue coverage of the face, muscles, fascias, and retaining ligaments**. b** Drawing after anatomical preparation of the zygomatic-cutaneous ligament**. c** The main retaining ligaments

**Fig. 7:**
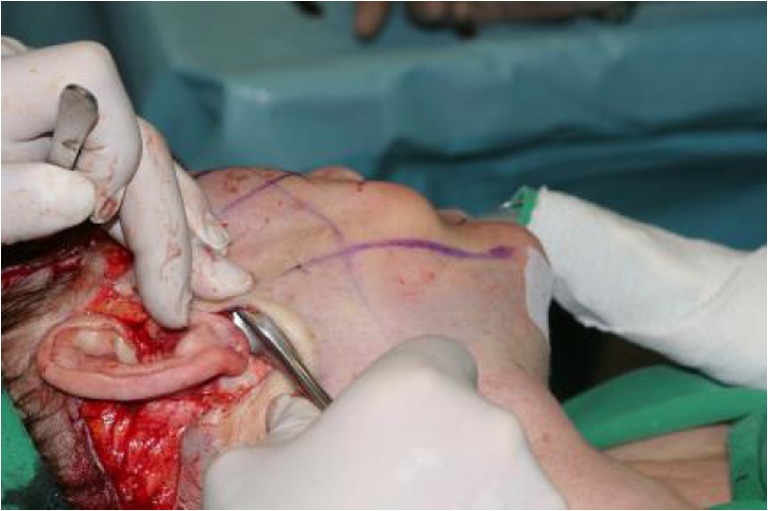
Distension of the cervical wing

**Fig. 8:**
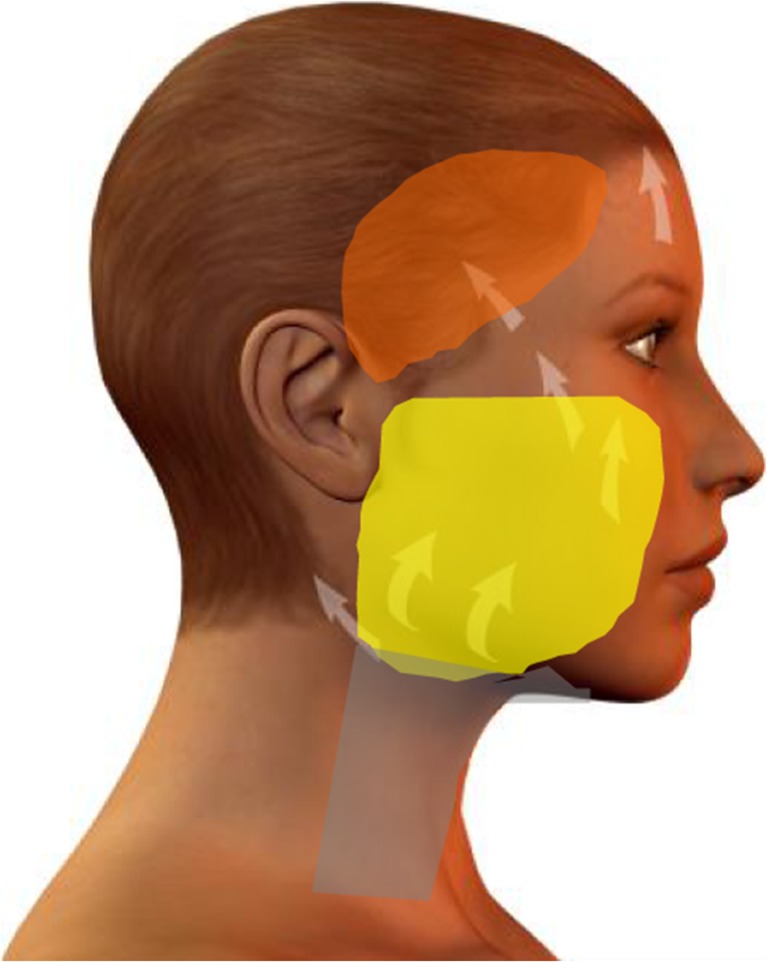
SMAS compartments: suprazygomatic, epizygomatic, infrazygomatic, and platysmal SMAS

**Fig. 9:**
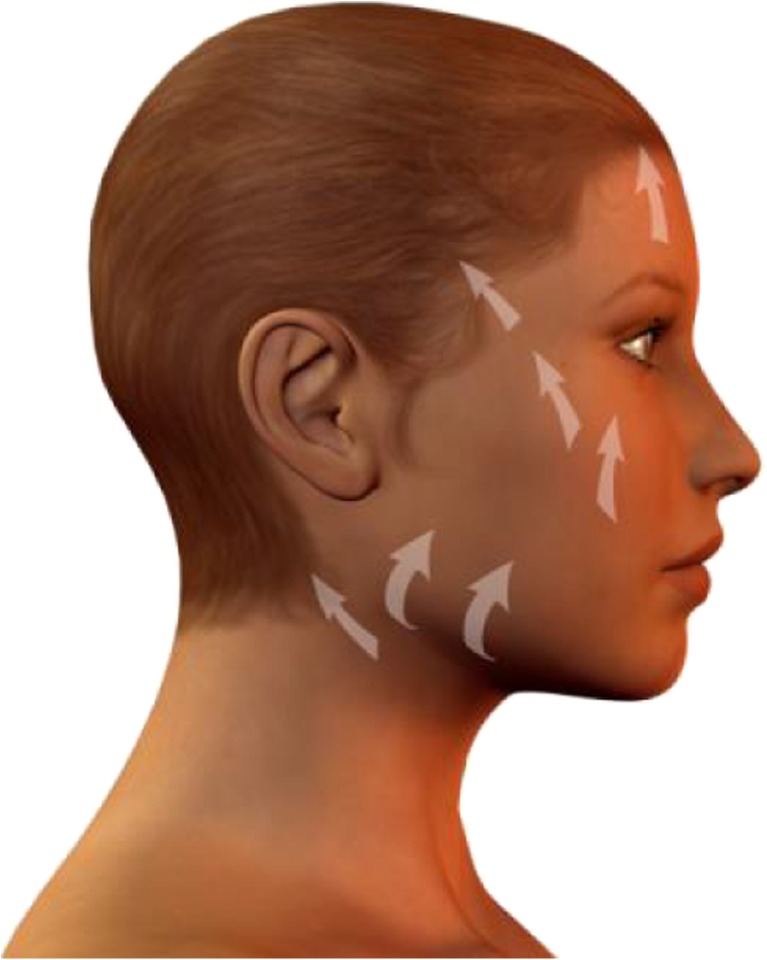
Subcutaneous vectors

We postulate that a number of key elements determine whether or not the central challenge of facelift surgery defined above will be met: Preservation of most retaining ligaments (Fig. 6a, b), as these serve as “guardians of identity” [23]^2^. It is often not widely enough appreciated that retaining ligaments show major interindividual and inter-ligament variations in elasticity and strength. Also, they serve to fix the facial soft tissue mass in loco, both in the resting state and during facial movements (mimics). Therefore, retaining ligaments are a crucial feature of individual esthetics. As a general rule, distend them carefully. One notable exception of a retaining ligament whose dissection produces the least negative facial expression effects may be the zygomatic-cutaneous ligament (Fig. 6c). Preservation of the SMAS intersegmental connections (Fig. 7). It must be kept in mind that facial subcutis is highly structured and compartmentalized into defined, cosmetically relevant anatomical zones (Fig. 2). If the intersegmental connections between these zones are preserved, both segmental compression and excessive dilatation effects are avoided (Fig. 8). This retains a major point of facial soft tissue fixture. Midfacial-submalar preparation in order to reduce aging-associated facial skelettalisation and lengthening of the lower eyelid and subplatysmal preparation and disconnection of the cranial-platysmal border allows one to stretch the horizontal neck angle into cranial direction, following the natural stretch lines of this region (Fig. 9), thereby permitting optimal modeling of neck structure. To obey these principles greatly facilitates to achieve not only facial rejuvenation but also an earlier, more youthful facial phenotype that the patient recognizes as “self.” The three-dimensional SMAS tethering technique introduced here strives for facial naturalness. This is achieved by sculpting the segmental sub-SMAS volume via tethering while simultaneously preserving the “guardians of facial individuality,” i.e., the retaining ligaments and the zygomatic SMAS border (PRESTO). Most recently, Basile et al. have reported an interesting technique that dissects the retaining ligaments and make a reduced tunneling release of the SMAS in order to facilitate upwards movement of the sub-SMAS volumetric tissue mass together with interconnecting structures [25]. However, this technique differs from the current one in that it does not employ the composite layer technique of Hamra [26], and therefore impacts less on the decisive midface and suborbital area. Also, the Basile et al. method does open the lateral SMAS border about 3 cm and thus entails possibilities of “overmobilization” and lateral overcorrection. Finally, in contrast to the PRESTO method, this technique does not enter into the subplatysmal area and does not release the mandibular margin. Therefore, this sensible technique elegantly underscores that it is indeed possible to achieve upwards movement of sub-SMAS tissue, but is much more limited than the PRESTO technique in its freedom of harmonic facial structuring, namely in the suborbital, midface, and neck region. Of note, the PRESTO technique allows a complete, essentially unlimited facial sculpting, while simultaneously providing an inbuilt safeguard mechanism against unnatural, identity-endangering facial overextension. Another advantage of the PRESTO technique, as compared to cutaneous lifting, lies in the sustainability of the surgical results: Since the SMAS is a firm, tissue-fixing, yet moderately flexible structure; it is being recruited to enhance tissue elasticity and stability. It is well-appreciated that subperiostal face-lifting techniques [27, 28] can generate an intense facial alienation phenotype (e.g., “trumpeting angel” phenomenon [29]) because of its tendency to produce unnatural tissue bulging. Also, subperiostal face-lifting does not allow one to move the distinct relevant tissue planes of the facial aging process (i.e., the subcutis and sub-SMAS area incl. the facial fat pads) in a well-coordinated, equidirectional manner moving exclusively the periost will produce both bulging and unnatural tightening. Moreover, since the primary zones of preparation with the current technique occur in so-called mobile layers/shunting layers that are only slightly vascularized, the novel face-lifting method reported here carries a minimal risk of injuries to nerves, blood vessels, parotid duct, and muscle structures.

## Conclusion

The further development of the primary SMAS technology according to Skoog [30] into a super extended rhytidectomy technique according to Hamra [31] or into a subperiostal technique according to Ramirez [22], with endoscopic support or the opening of the retaining ligaments according to Stuzin [32], shows that constant efforts are being made to detach and to fixate tissues anew, either with cutaneous SMAS preparation and marginal SMAS extension or from a subperiostal aspect using volume compression. These different techniques have in common that they all involve complete preparation and new fixation of margins. The combination of SMAS tethering and preservation of retaining ligaments introduced here as a novel, rapid, safe, and pragmatic new face-lifting technique (PRESTO) conserves the individual esthetics of the patient by approaching her/his individual facial characteristics from decades ago. At the same time, undesired standard outcomes of facelift surgery are avoided. The general concept that the natural facial boundaries and fixation points deserve to be respected is already reflected in the SMASectomy approach of Baker [33] and its modification by Graf [34], which is characterized by non-undermining of the SMAS and by vector limitation through the removed tissue section. This, however, may even aggravate the appearance of facial aging because of volume reduction. Instead, the current technique reported here repositions tissue volume (Fig. 1m).

## Footnotes

1 Terylene^®^ is a trade mark of ICI.

2 Prepared for submission in DERMATOLOGICAL SURGERY “Anatomical determinants of facial identity: The central importance of retaining ligaments and SMAS.”
